# The Structure of the Active Pd State During Catalytic Carbon Monoxide Oxidization

**DOI:** 10.1021/acs.jpclett.1c00620

**Published:** 2021-05-06

**Authors:** Christopher M. Goodwin, Mikhail Shipilin, Stefano Albertin, Uta Hejral, Patrick Lömker, Hsin-Yi Wang, Sara Blomberg, David Degerman, Christoph Schlueter, Anders Nilsson, Edvin Lundgren, Peter Amann

**Affiliations:** † Department of Physics, Stockholm University, 10691 Stockholm, Sweden; ‡ Synchrotron Radiation Research, Lund University, 22100 Lund, Sweden; § Photon Science, Deutsches Elektronen-Synchrotron (DESY), 22607 Hamburg, Germany; ∥ Department of Chemical Engineering, Lund University, 22100 Lund, Sweden

## Abstract

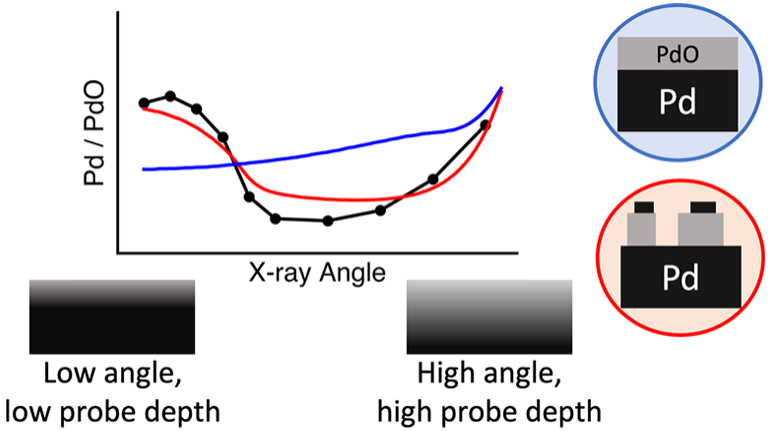

Using grazing incidence X-rays and X-ray photoelectron spectroscopy during the mass transfer limited catalytic oxidation of CO, the long-range surface structure of Pd(100) was investigated. Under the reaction conditions of 50:4 O_2_ to CO, 300 mbar pressure, and temperatures between 200 and 450 °C, the surface structure resulting from oxidation and the subsequent oxide reduction was elucidated. The reduction cycle was halted, and while under reaction conditions, angle-dependent X-ray photoelectron spectroscopy close to the critical angle of Pd and modeling of the data was performed. Two proposed models for the system were compared. The suggestion with the metallic islands formed on top of the oxide island was shown to be consistent with the data.

An essential aspect in developing more efficient catalytic processes is understanding the active phase of the catalyst. Directly probing the catalyst as the reaction occurs is necessary, since there can be significant restructuring due to the interaction with the reactants.^1−3^ To develop an understanding of heterogeneous catalysis, surface-sensitive techniques to measure the structure and chemical state must be employed.^4,5^ One of the most valuable measurements is ambient pressure X-ray photoelectron spectroscopy (AP-XPS) due to the ability to directly measure the chemical state of both the surface and the gas phase.^6^ The recent development of an ultra-high-vacuum to high-pressure (over 1 atm) AP-XPS allows for new pressure ranges to be utilized without the necessity of separate instruments or techniques.^7^ With the recent development of POLARIS by using directed gas flow, the first high-pressure XPS, it is now possible to bridge the pressure gap of traditional AP-XPS studies to the industrial scale pressures.^7^

Here, Pd(100) was used as a model catalyst to probe the structural rearrangement during CO oxidation approaching atmospheric pressures.^8−12^ The surface structure is of particular interest in Pd due to the possibility of surface reconstructions and oxide formation during the catalytic reaction.^13^ It has been well established that the metal oxidizes epitaxially with increasing oxygen affinity,^14−17^ but the reduction portion of the catalytic cycle is less understood. Scanning tunneling microscopy (STM) studies have suggested morphologic changes during the catalysis, and gas-phase measurements have shown that oscillations of catalytic activity are possible.^17−19^ The results of these studies indicate that the surface is dynamically changing under steady-state reaction conditions, and a more complex structure may appear as PdO becomes reduced. The complex structure of the reduced oxide may be highly active due to the mixed metal and oxide states.^20,21^

The dynamic nature of Pd(100) has limited surface morphology and chemical studies during reaction conditions to UHV or low pressures.^22−24^ Angle-resolved XPS, where the angle between the sample and analyzer is changed to determine a depth profile, is often challenging for AP-XPS due to geometric constraints. Furthermore, at the high kinetic energies required for high-pressure XPS, the electron mean free path (MFP) becomes quite long, making the information content more bulk sensitive and thereby lacking the essential surface information. These limitations make grazing incidence (GI) XPS an ideal electron spectroscopy tool to determine the surface compositions under different structural arrangements under realistic reaction conditions.^25,26^ POLARIS, a high-pressure XPS system, can achieve the reaction conditions of industrial catalysis, and by using X-rays at GI, the bulk-to-surface sensitivity can be varied allowing for depth profiles, opening the door to combined structural and chemical information using XPS at industrial conditions.

GI-XPS operates on the principle of total reflection, wherein below the critical angle, the light is reflected off the surface with a low penetration depth. At low angles, the X-ray light penetrates less deeply into the material; as the angle is increased, the penetration depth of the light increases. By calculating the penetration depth of the light and the escape likelihood of the photoelectrons, various models can be evaluated to determine the structure of the surface.

All measurements were taken with the POLARIS instrument^7^ at beamline P22^27^ of Petra III synchrotron at DESY, Hamburg. All spectra were gathered with 4600 eV photon energy using a double crystal monochromator of Si(311) with a beam divergence of 0.03°. Throughout the experiment, survey spectra were collected to track possible contaminations; at no point did any impurities appear.

Figure 1 shows an example of an angle-resolved Pd 3d_5/2_ spectrum. All XPS spectra were fitted with the CasaXPS ver. 2.3.23. The fitting included the Pd 3d_5/2_ metal peak, an asymmetric shape that incorporates the asymmetry expected for a metal peak, and the Pd CO 2√2×√2 adsorbed peak based on the pure metal, centered at 334.9 (±0.05) eV.^28,29^ For the oxide peak, a Gaussian–Lorentzian peak shape was used at 336.5 (±0.05) eV. The peak positions and assignments of the metal and oxide peaks are in good agreement with previous studies^30,31^ after correction for the recoil effect caused by the high-energy X-rays.^32^

**Figure 1:**
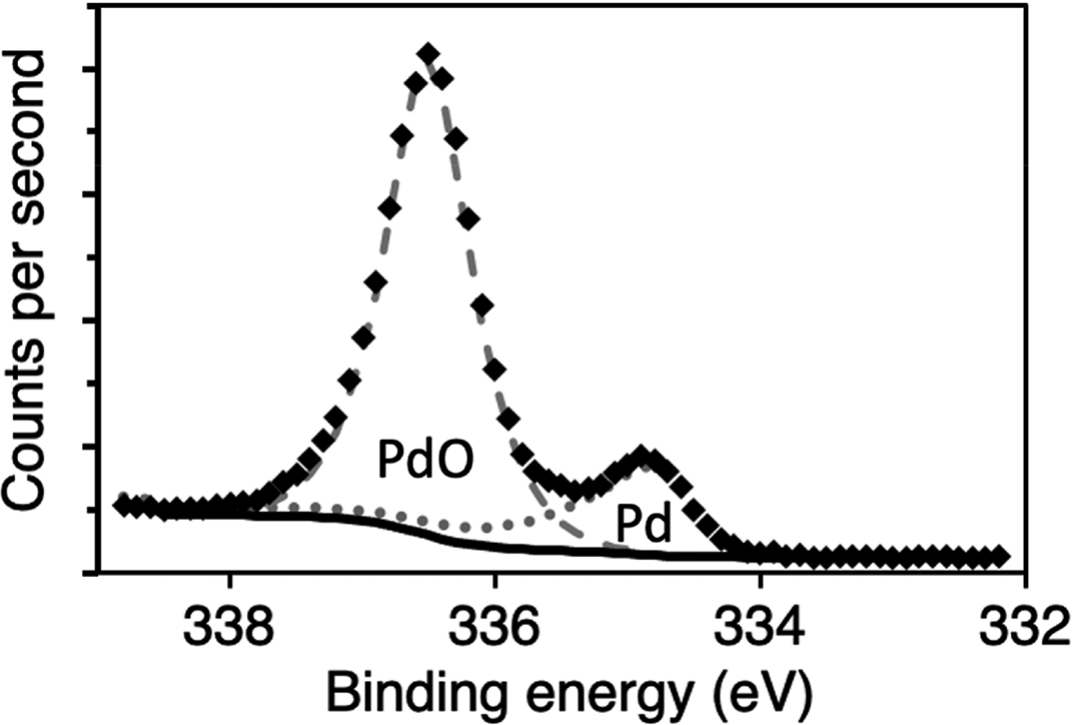
Example of peak fitting for the Pd 3d_5/2_ peak gathered after complete oxidation and partial reduction at 0.7° incidence, 300 mbar, and 360 °C. As described above, the spectra are fit with two components, a metal peak based on pure components shown with a dotted line and an oxide with a dashed line.

The oxidation and reduction of the sample were carried out at a total pressure of 300 mbar and a gas ratio of 50:4 O_2_ to CO. The pressure over the sample was determined by calibrating the first differential stage pressure with respect to the main chamber pressure; for more details, see Amann et al.^7^,^33^ The sample was first heated to 425 °C under reaction conditions, where the surface was covered with over 97% complete oxide. The sample was then cooled to 360 °C, where metal regions started to reappear. The sample was held at this temperature and pressure for several hours with no observable chemical change, indicating the system was at chemical equilibrium. The system was at the mass transfer limit (MTL) as measured by mass spectroscopy before the sample was oxidized, while the sample was an oxide, and at 360 °C.

Figure 2 shows the measured Pd/PdO intensity ratio as the incident angle of the X-rays relative to the surface is changed. The angle between the surface and the analyzer was held constant as in the experiment described elsewhere.^34,35^ When the incident angle of the photons approaches the critical angle, 0.7° for Pd, the X-ray field intensity within the sample changes. We clearly see a strong variation in the ratio as the angle is changed. At low angles, below 0.2°, there is a strong metal signal that rapidly diminishes as the angle increases forming a minimum in the ratio between 0.2° and 0.4°. At higher angles, the metal component then grows more rapidly again as for the very small angles.

**Figure 2:**
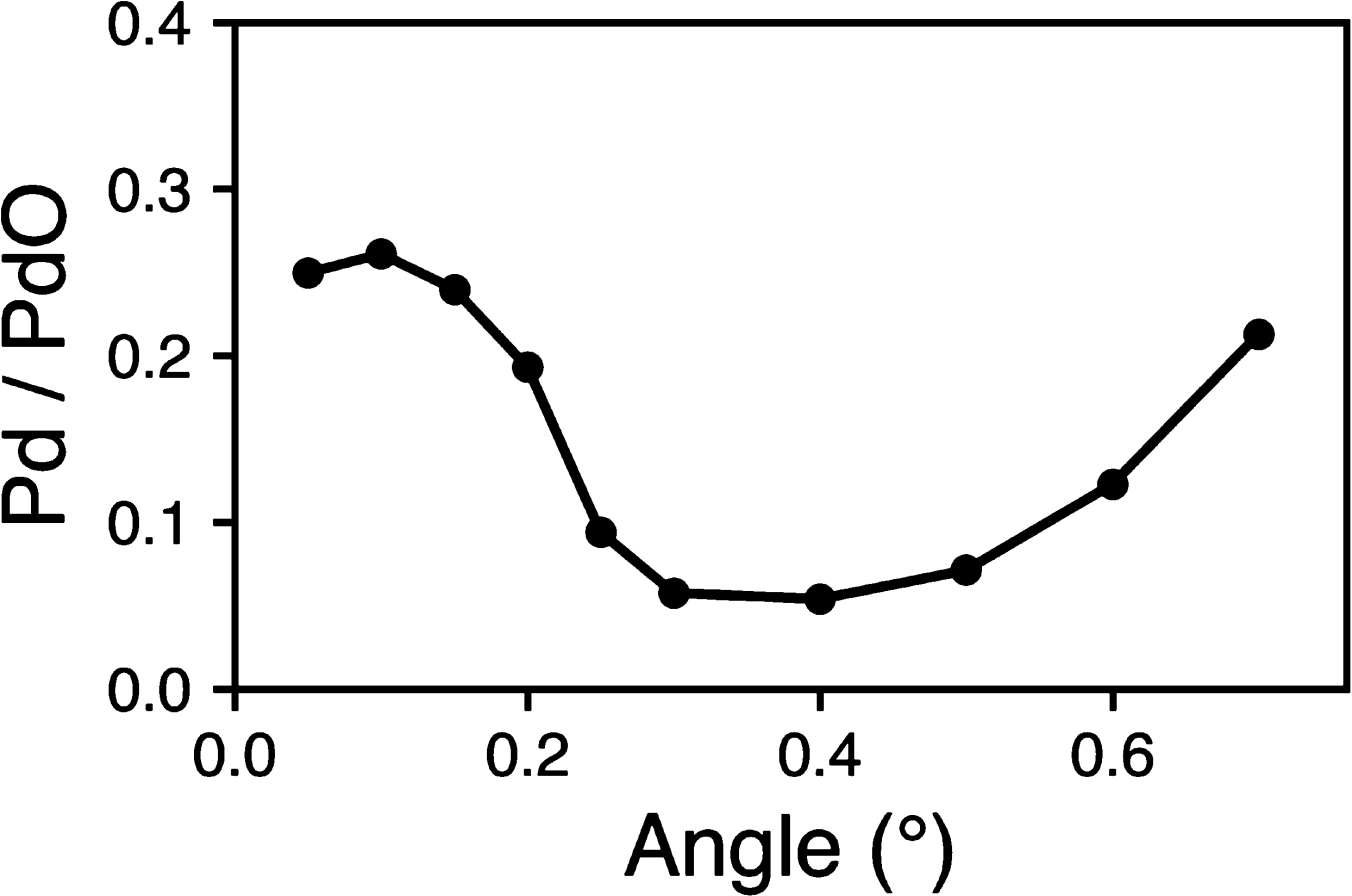
Ratio of metal to oxide peak intensity as a function of angle.

The bulk oxide is well-known to grow epitaxially in a Stranski-Krastanov mode exposing the PdO(101) surface, then to a bulk polycrystalline phase,^13^ but while the temperature decreases, there is an open question of how the metal reforms. There have been three observed processes for the reduction of palladium during the catalytic oxidation of CO under various reaction conditions, and herein we propose a fourth, all shown in Figure 3:

i. The oxide remains a complete oxide as the metal grows upon reduction from the bulk toward the surface.^36^
ii. The oxide remains a complete oxide as the metal grows from the surface toward the bulk, forming first small islands that thicken with layer by layer growth.^37^
iii. As the oxide is reduced, it forms a film of porous or island-like oxide structure, similar to how the oxide is formed.^38^
iv. The oxide is reduced from the surface inward and forms porous or island-like structures, a combination of (ii) and (iii).

**Figure 3:**
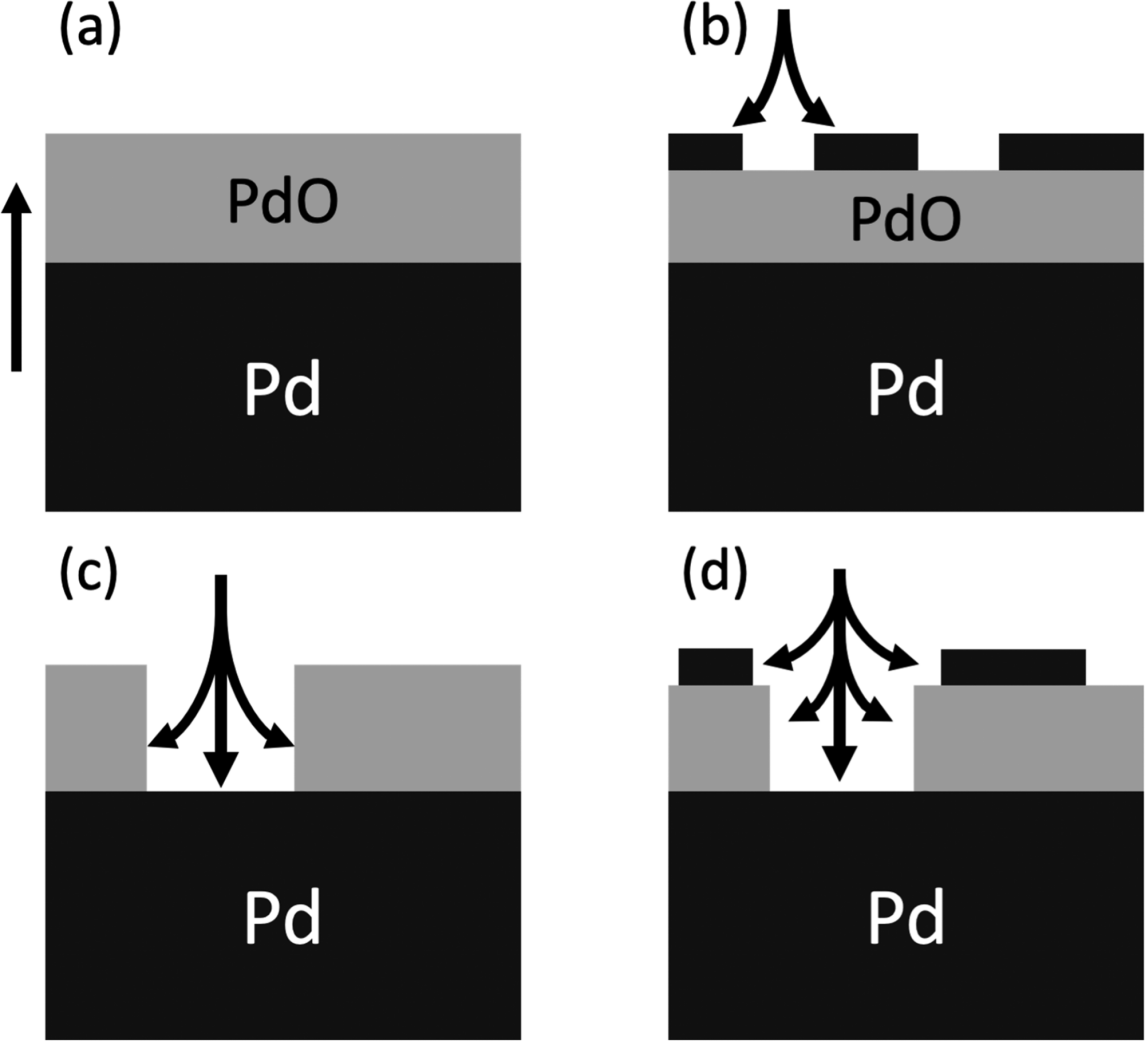
Simplified representation of the reduction processes of the four proposed hypotheses with gray representing PdO and black Pd. The arrows show how the surface rearranges as reduction occurs. Figure 4a corresponds to hypothesis (i) and so on.

These four hypotheses can be tested by measuring the metal to oxide signal ratio. At significantly high angles, all four hypotheses predict that the metal signal will dominate in intensity over the oxide. This is caused by higher angles generating a signal from deeper within the sample probing the bulk metal. The hypotheses that proposed a metal surface layer would predict higher metal signal at low angles, where the signal originates from topmost constituents before decreasing. Finally, hypotheses (iii) and (iv) propose an incomplete oxide film; this effect would allow the X-rays to penetrate more efficiently and for electrons to escape more easily.

By calculating the intensity of the field and including the effects of electron scattering through the different materials as a function of depth, a model can be constructed where the fitting parameters are the thicknesses of the metal and oxide layers (see SI for details).

The oxide film’s density was decreased from the bulk value to emulate the effects of a porous oxide of oxide islands like proposed in hypotheses (iii) and (iv). While this simplification accounts for the most considerable effect, the increased electron and photon permeability caused by depleted oxide films, but not changes to surface roughness (see SI for details).

Figure 4 shows the results of the four hypotheses fit to the experimental data. The solid line is the experiment data, while the dashed lines correspond to the various models, Figure 4a for hypothesis (i) and so on. The models’ qualitative trends match with the expectation being Figure 4c, which will be discussed later. Each of the four models provides quantitative information on the depth and effective density of the layers. Hypothesis (i) predicts an oxide layer of 27.7 Å, hypothesis (ii) an oxide layer 48.7 Å thick and a metal layer 1.1 Å thick, (iii) an oxide layer 40.0 Å thick with density 1.5 g/cm^3^, and (iv) an oxide layer 73.4 Å thick with density 1.1 g/cm^3^ and a metal layer 1.2 Å thick. The oxide layers’ thickness corresponds to between 5 and 15 layers depending on the model. The metal layer corresponds to a coverage of 0.4, mostly independent of the model used. The extremely low density indicates that the oxide film may not be in a 1 to 1 stoichiometric ratio with the Pd and may have island formation on the surface like those previously observed.^13^ Densities ranging from 0.1 g/cm^3^ to bulk density were evaluated (see SI for details). Other possible effects that were not included are layer mixing and morphologic effects such as roughness. All of the models assume a static surface; this may also contribute to the inaccuracy of the models. Dynamic changes in the local density of the surface would affect parameters such as MFP and X-ray permeability.

**Figure 4:**
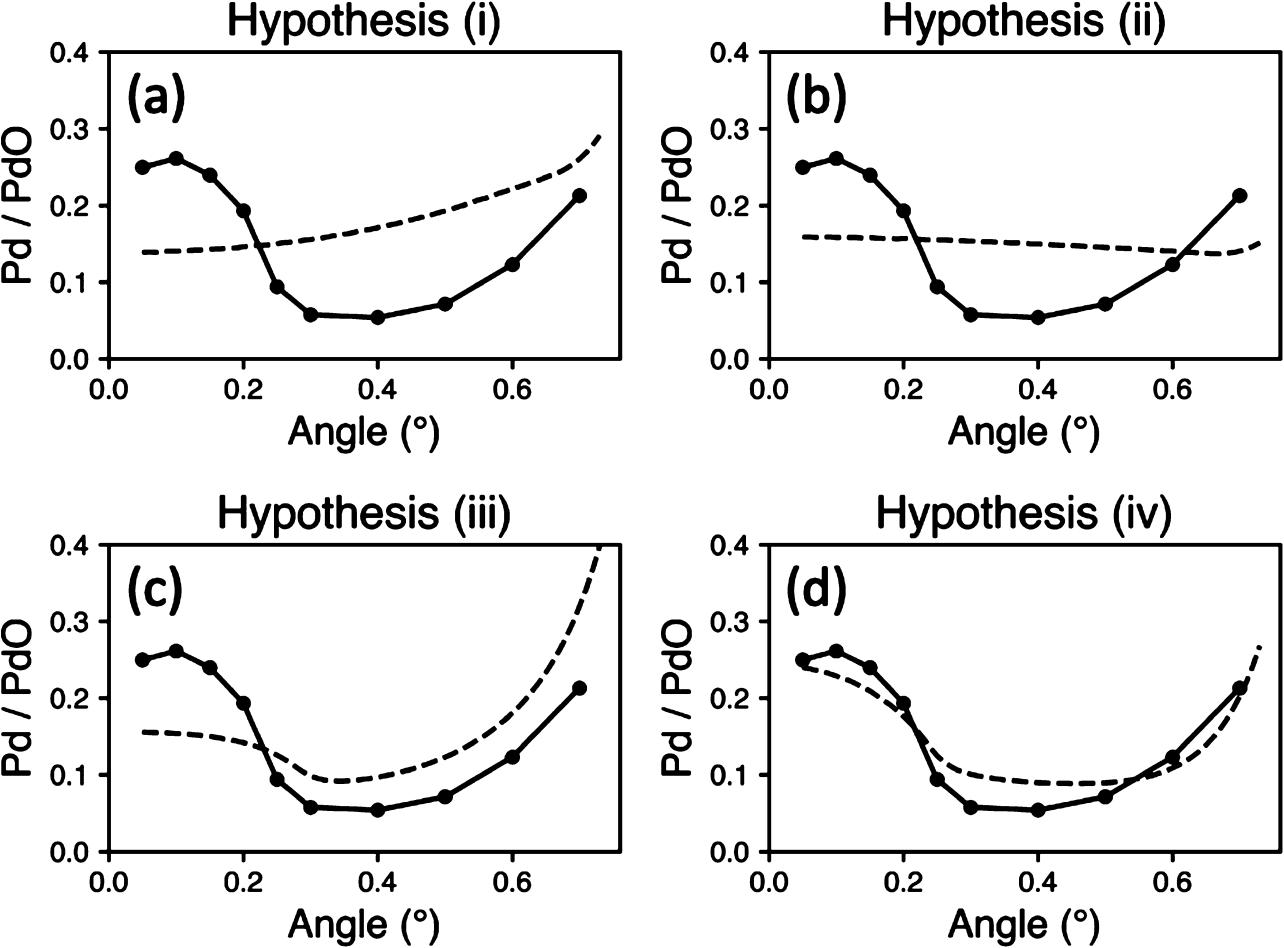
The four proposed hypotheses fit the experimental data. The solid line is the experimental data collected and is the same in all four figures. The dashed line represents the four hypotheses (a) for (i) and so on.

As mentioned previously, Figure 4c does not match the qualitative trend outlined before. The more complex shape of the simulated data is due to the effect of low densities on the MFP of electrons. The electrons generated in the very-low-density oxide are not heavily scattered until a significant amount of the oxide is probed. Therefore, the ratio of metal to oxide emulates the ratio of the electron MFP within the metal and oxide (see SI for details).

The structural changes during PdO reduction are of particular interest, as they are not widely understood and may differ significantly from other CO oxidation catalysts such as platinum. Previous studies have shown that Pt can form CO islands that are known to be highly active.^39^ The result of Pd island forming on top of PdO shows Pd similarity to Pt, as both have active metal island phases. A significant difference remains as PtO is not an active catalyst for CO oxidation,^40^ yet as described above, PdO is still an active catalyst by the fact that a 97% oxide surface remained within the MTL. The PdO(101) oriented oxide on the Pd(100) has previously been shown to be an active phase for CO oxidation,^16,20,22^ although the different activity of the metal and oxide is still being discussed. The reason for the active PdO(101) surface is the presence of undercoordinated Pd atoms at the surface, similar to the RuO_2_(110) and IrO_2_(110) surfaces,^41,42^ which may not be the case for PtO. Yet, in the present study, at no point was the sample a complete oxide; as a result, the catalytic activity may be due to the formation of metallic islands. However, more research would be needed to test this further.

## Supporting Information Available

MS setup; GI-XPS eqs and fitting procedure; Density model for island and porous oxides (PDF)
