# Supplementary material for: The Structure of the Active Pd State During Catalytic Carbon Monoxide Oxidization
Source: J Phys Chem Lett. 2021 May 6;12(18):4461–5. doi: 10.1021/acs.jpclett.1c00620 (PMC8279738; doi:10.1021/acs.jpclett.1c00620)
Supplement: Supplementary file 1 — jz1c00620_si_001.pdf [file jz1c00620_si_001.pdf]

The Structure of the Active Pd state During Catalytic Carbon Monoxide Oxidization

Christopher M. Goodwin[1], Mikhail Shipilin[1], Stefano Albertin[2], Uta Hejral[2], Patrick Lömker[3], Hsin-Yi Wang[1], Sara Blomberg[4], David Degerman[1], Christoph Schlueter[3], Anders Nilsson[1], Edvin Lundgren[2], Peter Amann[1]*

[1]Department of Physics, Stockholm University, 10691 Stockholm, Sweden

[2]Synchrotron Radiation Research, Lund University, 22100 Lund, Sweden

[3]Photon Science, Deutsches Elektronen-Synchrotron (DESY), 22607 Hamburg, Germany

[4]Department of Chemical Engineering, Lund University, 22100 Lund, Sweden

AUTHOR INFORMATION

Corresponding Author

* Christopher M. Goodwin – Department of Physics, Stockholm University, Stockholm 10691 Sweden. Email: Christopher.Goodwin@fysik.su.se

Mass spectroscopy set-up

Due to the high pressure in the main chamber, a residual gas analyzer in the main chamber is not an option; this is also true for the first stage of differential pumping. To this end, the mass spectra were gathered on a differentially pumped MS chamber attached to the backing pump line to the first stage turbomolecular pumps. Figure 1 shows a diagram of the position of the MS relative to the reaction environment. To control the MS pressure, a leak valve is set to a given open position such that the maximum pressure of the experiment does not exceed the operating pressure of the MS. This means that for sets of experiments where pressure is changed, the relative signal of the MS varies as well. The effect of changes in the absolute signal can be accounted for by monitoring the pressure in front of the MS or using a trace inter gas like helium to normalize the MS signal.

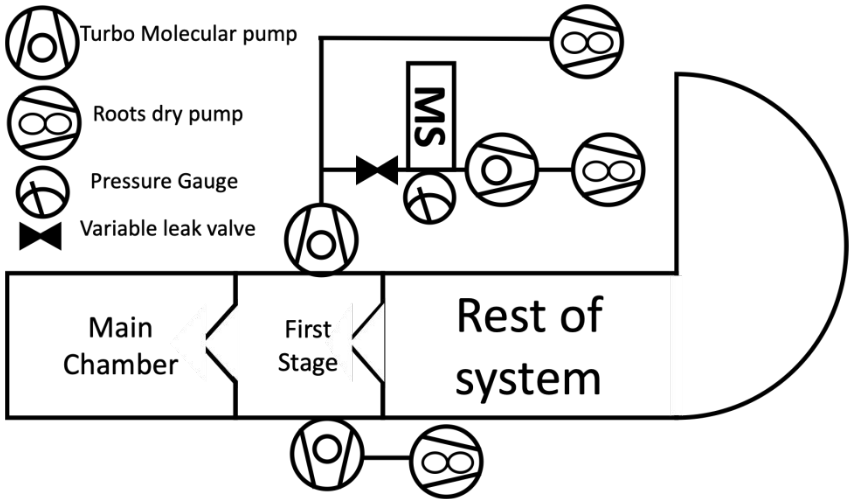

Figure 1. A simplified diagram of the AP-XPS system showing the placement of the MS used for all the experiments.

GI-XPS Equations

To predict the XPS intensity, the well-known equations 1 and 2 were used.[1]

$$\mathrm{I_o} = n\,f\,\sigma\,\varphi\,y\,\lambda'\,A\,\Psi\,e^{-PoS/\mathrm{kT}} \qquad (1)$$

$$\mathrm{I} = \mathrm{I_o} e^{-d/\lambda} \qquad (2)$$

Where I_o in equation 1 is the photon intensity of the pure material and I in equation 2 is the electron intensity in the resulting XPS signal.

Starting with equation 1, n is the atomic density, f is flux, σ is the cross-section, φ is the angular acceptance, y is the ionization probability, λ' is the probe depth, A is the area illuminated by the x-rays, and Ψ is transmission through the analyzer. The last two terms describe the gas phase scattering due to the high pressure between the sample and the detector and the electron scattering within the bulk material. Variables P, o, and S correspond to pressure, gas-phase scattering cross-section, and separation between the sample and vacuum. Terms k and T have the ordinary meaning of the Boltzmann constant and temperature.

To calculate the intensity of the resulting XPS signal in equation 2, d is the depth of electrons through the bulk, the mean free path values λ were calculated by the TPP-2M model.[2]

For the present measurements, by taking the ratio of Pd to PdO in the Pd $3d_{5/2}$ region σ, φ, y, A, and Ψ in equation 1 cancel in equation 2, see equation 3 where the crossed out terms cancel, herein the summation is over the depth that the material exists. The gas-phase scattering terms cancels since the PdO and Pd core level peaks are very similar in kinetic energy and therefore are attenuated by the gas phase at the same rate. Though in the case where kinetic energies are very different gas-phase scattering should not be neglected; see Karwasz et al. for more details.[3]

$$\frac{\mathrm{I}_{\mathrm{Pd}}}{\mathrm{I}_{\mathrm{PdO}}} = \frac{\sum n_{Pd}\, f_{Pd}\, \cancel{\sigma_{Pd}}\, \cancel{\varphi}\, \cancel{y_{Pd}}\, \lambda'_{Pd}\, \cancel{A}\, \cancel{\Psi}\, \cancel{e^{-\frac{PoS}{\mathrm{kT}}}}\, e^{-\frac{d}{\lambda_{Pd}}}}{\sum n_{PdO}\, f_{PdO}\, \cancel{\sigma_{Pd}}\, \cancel{\varphi}\, \cancel{y_{Pd}}\, \lambda'_{PdO}\, \cancel{A}\, \cancel{\Psi}\, \cancel{e^{-\frac{PoS}{\mathrm{kT}}}}\, e^{-\frac{d}{\lambda_{PdO}}}} \qquad (3)$$

While under typical angle-resolved measurements, the x-ray flux I_0 also cancels but at angles close to the critical angle, an evanescence or standing wave of the incident photons form at the surface, strongly enhancing the photon intensity from the surface. As a result, the photon flux is very sensitive to the incident angle and the resulting probing depth. To determine the resulting x-ray intensity close to the critical angle, the Sergey Stepanov x-ray server was used.[4,5] As a result of the critical angle phenomena, λ' needs to be calculated for each angle, not necessary using non-grazing incidence XPS. Further, care must be taken for the correct polarization, herein the x-rays were 99% p-polarized, and the Henke data was used.[6] No

roughness nor transition region was included in these calculations.

Using the model of Pd bulk coated with a PdO film, coated with metallic islands at the surface of the PdO film a more complete version of equation 3 can be constructed. All the sources of metal signal appear in the numerator, while the oxide appears in the denominator.

$$\frac{I_{Pd}(\Theta)}{I_{PdO}(\Theta)} = \frac{\begin{pmatrix} \sum_0^d e^{-x/\lambda_{Pd}} f(x,\Theta)\, n_{Pd}\, \lambda'_{Pd} \\ + \sum_k^\infty e^{-x/\lambda_{Pd}} f(x,\Theta)\, n_{Pd}\, \lambda'_{Pd} \end{pmatrix}}{\sum_d^k e^{-x/\lambda_{PdO}} f(x,\Theta)\, n_{PdO}\, \lambda'_{PdO}} \qquad (4)$$

By this approach, equation 4 is calculated for the model presented in the main text, where d is the thickness of the metal surface and k the thickness of the oxide, and Θ is the incident x-ray angle. The second model of a porous oxide is represented by equation 5, where l is the oxide layer's thickness.

To perform probe the depth-profile of the surface, the X-rays' incident angle was changed about the critical angle of reflection from 0.05° to 0.70°. The resulting ratio of metal to oxide XPS components was then used to determine the thickness and location of the metal or oxide layers following the method

outlined by elsewhere.[7,8] This was done by solving equations 6 and 7 for d, k, and l at all angles, then using the result to refine the model. From there, the x-ray field is recalculated, and equations 4 and 5 are solved again. This is repeated until there is no change in the solution of thicknesses to within 0.1 Å. The code used to all calculations can be provided given a reasonable request is made.

$$\frac{I_{Pd}(\Theta)}{I_{PdO}(\Theta)} = \frac{\sum_0^l e^{-x/\lambda_{PdO}} f(x,\Theta)\, n_{PdO}\, \lambda'_{PdO}}{\sum_l^\infty e^{-x/\lambda_{Pd}} f(x,\Theta)\, n_{Pd}\, \lambda'_{Pd}} \qquad (5)$$

GI-XPS fitting procedure

Figure 2 shows a diagram of how the fitting procedure was done. In essence an initial condition was chosen, and the x-ray field was calculated for all angles and at all depths. From there the XPS intensities were calculated with the given layered structure and thickness by the equations above. With the same x-ray field, a new thickness for the layers was estimated and the relative error of the model was evaluated. The refinement of the thicknesses of the layers, with the same x-ray field, was iterated until the error of the model reached a minimum. From there a new x-ray field was calculated and the processes repeated. This was continued until the model no longer changed to within 0.1Å from the x-ray field.

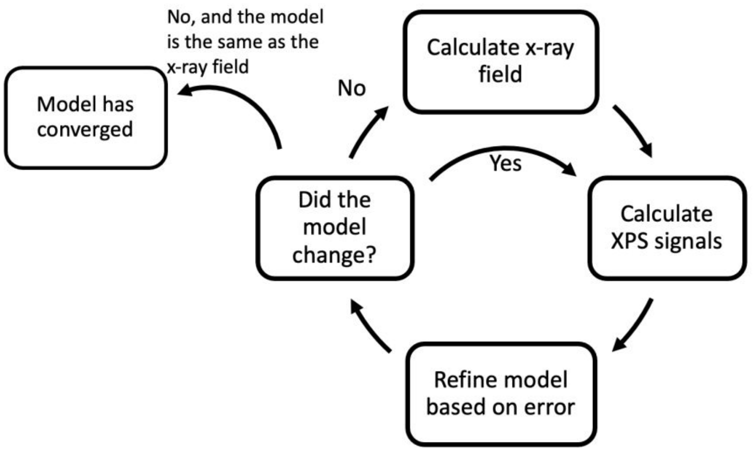

Figure 2. A flow chart to represent the modeling process.

Density model for island and porous oxides

To model the porous oxide film, a simplified model was used. A film of uniform and lower density was used to emulate the effects of a porous oxide, or oxide islands. The density of the oxide was perturbated from the bulk value of 8.3 g/cm^3. To model different densities a given density is chosen, converted to atomic density the electron mean free path was recalculated as well as probe then following the procedure described above. The thicknesses were calculated for a wide range of densities, from 0.1-7.3 g/cm^3. Figure 2 shows the resulting quality of fit for all the densities calculated in root mean squared(RMS) error. It should be noted that at densities far below what is physically possible, the simulations do not produce meaningful results. The low density maintains physical meaning based on the published work of Shipilin et al.[9]

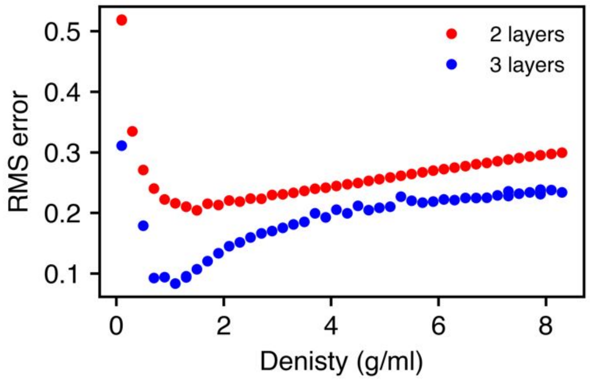

Figure 3. The trend of the RMS error between the simulation and the XPS data as a function of density.

It should be noted that the results from simulations with very low densities are not monotonic as would be expected. This results from the very low critical angle and a substantial mean free path of the signal. As seen in equations 2 and 3, the ratio of λ' become a scalar of any solution, and since at very low densities the λ' of the oxide changes at a much lower angle than the λ' of the metal, the simulated results follow the curve of the ratio of the λ' values. At higher densities, the metal and oxide's critical angle and material properties (and thereby the λ') are more similar; thus, the resulting simulation becomes monotonic. Figure 3 shows how the λ' of the oxide changes as a function of density.

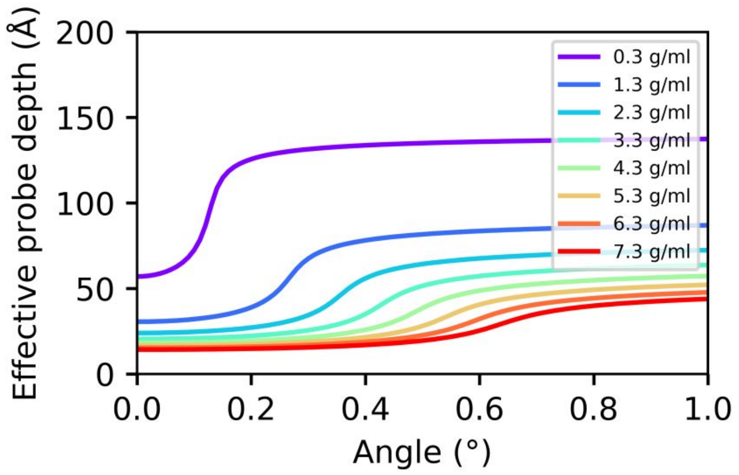

Figure 4. The effective probe depths (λ') of the oxide layer as a function of density and angle. As the oxide becomes less dense, the probe depth increases, and the critical angle decreases. This combination is the reason that low-density oxide films are not monotonic.

REFERENCES

(1) Moulder, J. F.; Stickle, W. F.; Sobol, P. E.; Bomben, K. D. Handbook of X-Ray Photoelectron Spectroscopy: A Reference Book of Standard Spectra for Identification and Interpretation of XPS Data; Physical Electronics Division, Perkin-Elmer Corporation, Eden Prairie 1992.

(2) Standard Reference Data, N. NIST Standard Reference Database 71. 1999.

(3) Karwasz, G.; Brusa, R. S.; Gasparoli, A.; Zecca, A. Total Cross-Section Measurements for e--CO Scattering: 80-4000 EV. Chem. Phys. Lett. 1993, 211, 529–533.

(4) Sergey Stepanov’s X-ray Server https://x-server.gmca.aps.anl.gov/ (accessed Jul 31, 2020).

(5) Stepanov, S. A. X-Ray Server: An Online Resource for Simulations of x-Ray Diffraction and Scattering. In Advances in Computational Methods for X-Ray and Neutron Optics; SPIE: Denver, 2004, 5536, 16–26.

(6) Henke, B. L.; Gullikson, E. M.; Davis, J. C. X-Ray Interactions: Photoabsorption, Scattering, Transmission, and Reflection at E = 50-30, 000 EV, Z = 1-92. At. Data Nucl. Data Tables 1993, 54, 181–342.

(7) Jach, T.; Gormley, J.; Thurgate, S. Grazing Incidence X-Ray Photoemission Spectroscopy of SiO2 on Si. Spectrochim. Acta, Part B 1999, 54, 1539–1544.

(8) Mehta, M.; Fadley, C. S. Angular-Dependent X-Ray Photoemission Study of Oxidized Silicon at Low X-Ray Incidence Angles. Chem. Phys. Lett. 1977, 46, 225–230.

(9) Shipilin, M.; Gustafson, J.; Zhang, C.; Merte, L. R.; Stierle, A.; Hejral, U.; Ruett, U.; Gutowski, O.; Skoglundh, M.; Carlsson, P. A.; Lundgren, E. Transient Structures of PdO during CO Oxidation over Pd(100). J. Phys. Chem. C 2015, 119, 15469–15476.
